# Characterization of the complete chloroplast genome sequence of *Fritillaria delavayi*, an ethnomedicinal plant in China

**DOI:** 10.1080/23802359.2019.1674742

**Published:** 2019-10-10

**Authors:** Peng Che, Xueping Wei, Yichen Song, Bengang Zhang, Haitao Liu, Yaodong Qi

**Affiliations:** a Key Laboratory of Bioactive Substances and Resources Utilization of Chinese Herbal Medicine, Ministry of Education, Institute of Medicinal Plant Development, Chinese Academy of Medical Sciences, Peking Union Medical College, Beijing, China;; b Engineering Research Center of Tradition Chinese Medicine Resource, Ministry of Education, Institute of Medicinal Plant Development, Chinese Academy of Medical Sciences, Peking Union Medical College, Beijing, China

**Keywords:** *Fritillaria delavayi*, chloroplast genome, phylogenetic analysis

## Abstract

*Fritillaria delavayi* has been widely used as a traditional Chinese medicine (TCM) to treat respiratory diseases for thousands of years. In this study, the complete chloroplast genome of *F. delavayi* was assembled. The circular genome is 151,938 bp in size, which is comprised of one large single-copy (LSC) region of 81,757 bp and one small single-copy (SSC) region of 17,537 bp and separated by a pair of inverted repeat (IR) regions of 26,322 bp. A total of 112 unique genes (78 protein-coding, 30 tRNA, and 4 rRNA) are predicted and 19 of them are duplicated in IR regions. The overall GC content is 37.0% while the GC content of the LSC, SSC, and IR regions are 34.8, 30.5, and 42.5%, separately. Phylogenetic analysis indicated that *F. delavayi* was closely related to *F. cirrhosa*.

*Fritillaria delavayi* Franch. (Liliaceae) mainly distributes in sandy and gravelly places in the Qinghai-Tibet Plateau and its adjacent areas. The bulbus of *F. delavayi* is one of the most commonly-used antitussive and expectorant in China, Japanese, Korea and many other countries (Zhou et al. 2010). It is officially recorded in the Chinese Pharmacopoeia as one source of ‘Chuan bei mu’ (National Pharmacopoeia Committee 2015). The mainly phytochemical constituents of *F. delavayi* are alkaloids, saponins, terpenoids, etc. Pharmacological studies have proven alkaloids to be the most bioactive and potent components (Li et al. 1999). In the present study, the complete chloroplast (cp) genome of *F. delavayi* was determined by using Illumina paired-end sequencing data.

In this study, the fresh leaves of *F. delavayi* are collected from Qinghai province of China (102°0'16″E, 36°59'48″N) and the voucher specimen (QHHZ20190525) was stored in the herbarium of Institute of Medicinal Plant Development, Chinese Academy of Medical Sciences, Peking Union Medical College (IMD). The total genomic DNA was isolated according to the manufacturer’s instructions of the Plant Genomic DNA Kit (Tiangen Biotech, Beijing, China) and subsequently sequenced by Illumina HiSeq platform with a PE150 genomic library. BLAST++ was used to filter the cp genomic reads and SOAPdenovo2 and SSPACE-STANDARD3.0 were applied to assemble and extend the complete cp genomes (Boetzer et al. 2011; Luo et al. 2012) with that of its congener *F. hupehensis* (GenBank accession number KF712486) as the initial reference genome (Li et al. 2014). The annotated genomic sequence has been submitted to GenBank with the accession number MN480806.

The chloroplast genome of *F. delavayi* was 151,938 bp in size, which is the typical circular quadripartite structure of angiosperm cp genomes and consists of one LSC region of 81,757 bp and one SSC region of 17,537 bp and separated by a pair of IR regions of 26,322 bp. A total of 112 unique genes (78 protein-coding, 30 tRNA, and four rRNA species) were predicted and 19 of them are duplicated in IR regions. Among these genes, 16 genes (10 protein-coding genes and 6 tRNA genes) had a single intron while two protein-coding genes (*clp*P and *ycf*3) had two introns. The total GC content is 37.0%, while the corresponding values of the LSC, SSC, and IR regions are 34.8, 30.5, and 42.5%, respectively.

Both Bayesian inference (BI) and maximum likelihood (ML) were performed in MrBayes v3.2.1 and CIPRES Science Gateway (http://www.phylo.org) respectively (Miller et al. 2010; Ronquist et al. 2012) to evaluate the phylogenetic position of *F. delavayi* in Liliaceae. *Colchicum autumnale* was chosen as the outgroup. The results showed *F. delavayi* was the sister of *F. cirrhosa* with high support (Figure 1). The complete cp genome of *F. delavayi* could be used as a super barcode to distinguish *Fritillaria* species and also to gain deeper insights into the promising ethnomedicinal plant.

**Figure 1.**
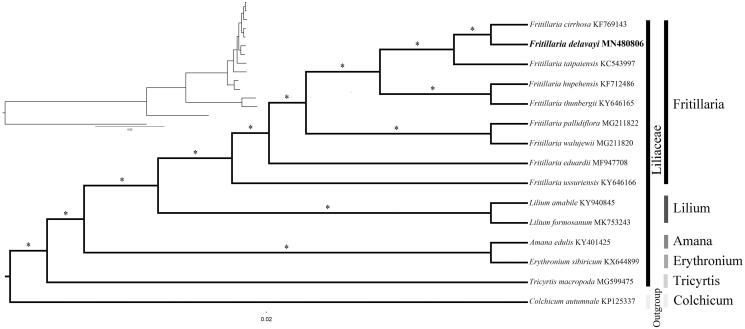
Phylogeny of 14 species within Liliaceae based on Bayesian inference (BI) of the concatenated chloroplast protein-coding sequences. Relative branch lengths are shown at the top-left corner. ‘*’ above the branches indicate that bootstrap support values of ML equal 100% and Bayesian posterior probabilities equal 1.0. Accession numbers are shown after the species names.
